# Empagliflozin targets Mfn1 and Opa1 to attenuate microglia-mediated neuroinflammation in retinal ischemia and reperfusion injury

**DOI:** 10.1186/s12974-023-02982-9

**Published:** 2023-12-12

**Authors:** Zhenlan Yang, Yidan Liu, Xuhao Chen, Shaofen Huang, Yangyang Li, Guitong Ye, Xu Cao, Wenru Su, Yehong Zhuo

**Affiliations:** State Key Laboratory of Ophthalmology, Zhongshan Ophthalmic Center, Sun Yat-Sen University, Guangdong Provincial Key Laboratory of Ophthalmology and Visual Science, Guangzhou, 510060 China

**Keywords:** Retinal ischemia and reperfusion injury, Neuroinflammation, Empagliflozin, Mitofusin 1, Optic atrophy 1

## Abstract

**Background:**

Neuroinflammation and mitochondrial dysfunction play crucial roles in retinal ischemia and reperfusion (IR) injury. Recent studies have identified mitochondrial function as a promising target for immunomodulation. Empagliflozin (EMPA), an anti-diabetic drug, has exhibited great potential as both an anti-inflammatory agent and a protector of mitochondrial health. This study aimed to assess the therapeutic efficacy of EMPA in retinal IR injury.

**Methods:**

To evaluate the protective effects of EMPA, the drug was injected into the vitreous body of mice post-retinal IR. Single-cell RNA sequencing (scRNA-seq) analysis was conducted to uncover the underlying mechanisms, and the results were further validated through in vivo and in vitro experiments.

**Results:**

EMPA effectively protected retinal ganglion cells (RGCs) from IR injury by attenuating local retinal inflammation. The scRNA-seq analysis revealed that EMPA downregulated the nucleotide-binding domain and leucine-rich repeat containing protein 3 (NLRP3) signaling pathway and restored mitochondrial dynamics by upregulating the expression of mitochondrial fusion-related genes, Mitofusin 1 (Mfn1) and optic atrophy 1 (Opa1). These findings were further corroborated by Western blotting. In vitro experiments provided additional insights, demonstrating that EMPA suppressed lipopolysaccharide (LPS)-induced cell inflammation and NLRP3 inflammasome activation. Moreover, EMPA enhanced mitochondrial fusion, neutralized mitochondrial reactive oxygen species (mtROS), and restored mitochondrial membrane potential (MMP) in BV2 microglia. Notably, genetic ablation of Mfn1 or Opa1 abolished the anti-inflammatory effects of EMPA.

**Conclusions:**

Our findings highlight the positive contribution of Mfn1 and Opa1 to the anti-inflammatory therapeutic effect of EMPA. By restoring mitochondrial dynamics, EMPA effectively mitigates microglia-mediated neuroinflammation and prevents RGC loss in retinal IR injury.

**Supplementary Information:**

The online version contains supplementary material available at 10.1186/s12974-023-02982-9.

## Background

Characterized by irreversible retinal ganglion cells (RGCs) degeneration, retinal ischemia and reperfusion (IR) constitute the pathological mechanisms of various vision-threatening diseases, such as diabetic retinopathy and glaucoma [1, 2]. In glaucomatous eyes, increased intraocular pressure (IOP) restricts retinal blood supply and triggers a self-reinforcing destructive cascade to RGCs, which is further exacerbated by subsequent reperfusion [3, 4]. However, current treatments targeting retinal IR injury have yielded unsatisfactory results, necessitating the exploration of the pathogenesis and potential treatments from a novel perspective.

Neuroinflammation and mitochondrial dysfunction are well-established hallmarks of neurodegenerative diseases [5, 6]. Microglia, the most abundant resident immune cells in the retina, quickly become reactive following retinal IR injury and release multiple inflammatory mediators, rendering RGCs more susceptible [7, 8]. Our previous studies also highlighted microglia-mediated neuroinflammation as an important regulatory target in retinal IR injury [9–11].

Recent evidence suggests that therapies targeting mitochondrial health hold promise for attenuating neuroinflammation [12–14]. Mitochondrial dysfunction could not only directly lead to axonal degeneration and neuronal apoptosis, but also amplify neuroinflammation, creating a vicious circle. Mitochondria house a cache of mitochondrial damage-related model molecules (mtDAMPs), such as mitochondrial DNA (mtDNA), mitochondrial reactive oxygen species (mtROS), and adenosine triphosphate (ATP). During mitochondrial dysfunction, these mtDAMPs can be exposed to the cytoplasm or extracellular matrix, sparking a range of inflammatory responses. The release of these mtDAMPs activates nucleotide-binding domain and leucine-rich repeat containing receptors (NLRs), Toll-like receptors (TLRs), and cGAS-STING, thereby promoting the production of inflammatory cytokines, chemokines, and reactive oxygen species [15, 16]. Healthy mitochondria maintain in a dynamic balance of fusion and fission to sustain normal cellular morphology and function, while pathological conditions can shift this balance toward net mitochondrial fission [17]. The release of these fragmented mitochondria upregulates immunostimulatory mitochondrial molecules and then activates proinflammatory pathways [18, 19]. Thus, restoring mitochondrial function represents an effective therapeutic target for neuroinflammation and neurodegeneration.

Empagliflozin (EMPA), a sodium-glucose-cotransporter-2 (SGLT-2) inhibitor, is a novel hypoglycemic agent that reduces blood glucose levels by inhibiting glucose reabsorption in the renal tubule [20]. Large clinical trials have demonstrated that EMPA treatment reduced the risk of cardiovascular events, heart failure, and cognitive impairment, which are largely independent of improved glycemic parameters [21–23]. Animal experiments and further mechanistic research have also confirmed that EMPA exerts cardioprotective and neuroprotective effects by alleviating inflammation and oxidative stress [24, 25]. The anti-inflammatory effect of EMPA can be attributed to the reduction of proinflammatory cytokines, M2 macrophage polarization, as well as the diminished activity of NF-kB, NLRP3 and MyD88-related pathways [26–28]. Regarding mitochondrial biology, EMPA has been found to modulate mitochondria dynamics and metabolism, activate mitophagy, reduce mitochondrial DNA damage, and stimulate mitochondrial biogenesis [29–32]. Given this, it is reasonable to hypothesize that EMPA can attenuate mitochondrial dysfunction and inflammatory process triggered by retinal IR.

Therefore, this study aimed to investigate the therapeutic effect of EMPA on retinal IR injury and explore whether its protection of RGCs was associated with the inhibition of microglia-mediated neuroinflammation and regulation of mitochondrial dysfunction.

## Material and methods

### Retinal IR model and drug administration

C57BL/6J male mice (6–8 weeks of age) were purchased from the Guangdong Medical Laboratory Animal Center and housed in a Specific Pathogen Free animal facility at the Zhongshan Ophthalmic Center. The experimental procedures were approved by the Institutional Animal Care and Use Committee of the Zhongshan Ophthalmic Center (approval number: Z2022044).

The retinal IR models were established as described previously [33, 34]. Prior to the operation, mice were anesthetized with 1% pentobarbital sodium injected intraperitoneally. We carefully inserted a 30G needle connected to a normal saline (NS) bottle into the anterior chamber of the mice’s eyes. IOP was increased to 110 mmHg by elevating the NS bottle to a height of 150 cm and maintained for 1 h. Intravitreal injections of the drug were performed immediately after the IR model was established. A 2 μl volume of empagliflozin (100 μg/ml, Selleck, USA) or control vehicle (phosphate-buffered saline, PBS) was slowly injected into the intravitreal cavity using a Hamilton syringe fitted with a 32-gauge microneedle.

**Fig. 1:**
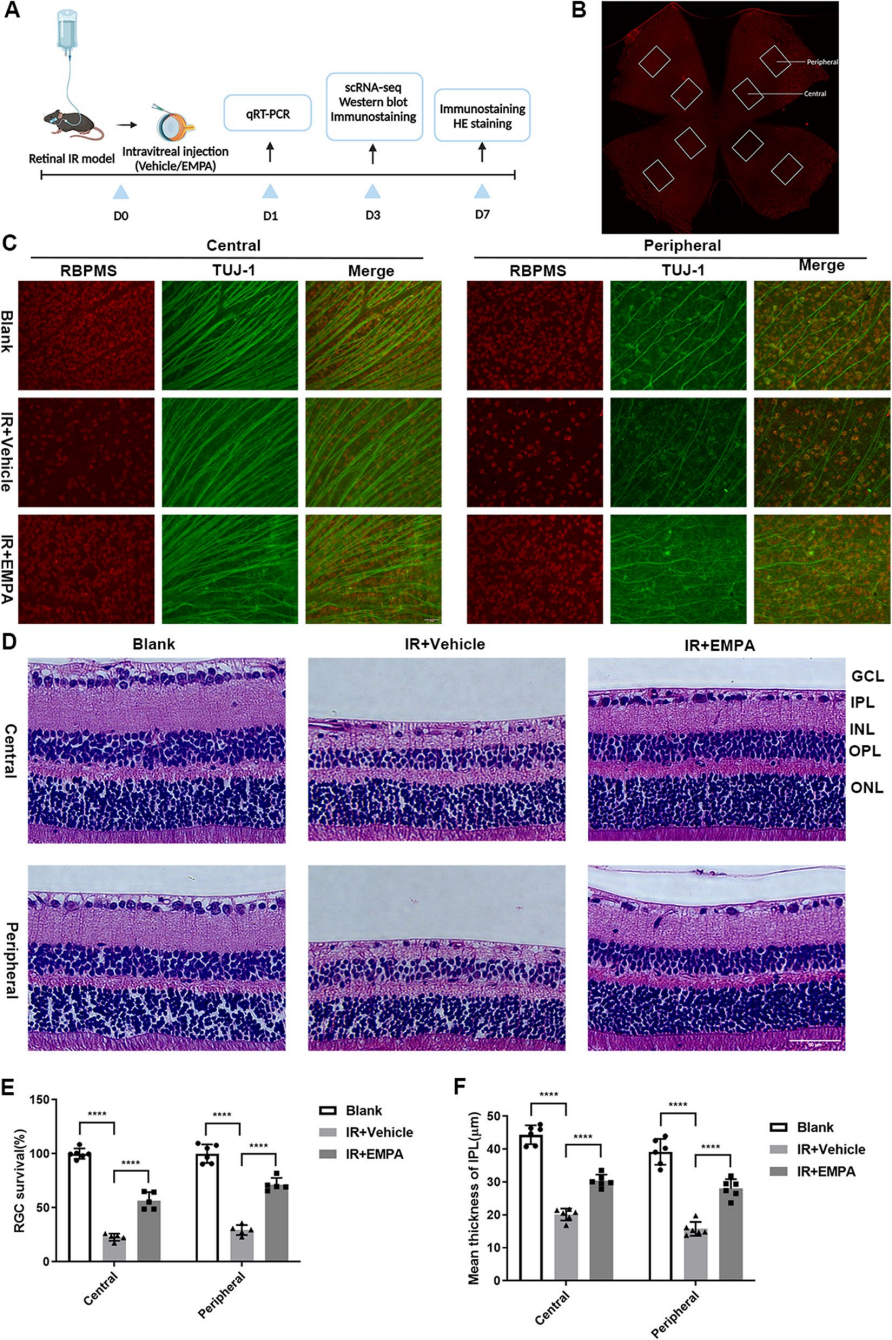
EMPA performs neuroprotective effects in the retinal IR model. **A** A schematic diagram of the timeline of IR induction, intravitreal injection treatment, and subsequent analyses. **B** Representative whole‐mount retina stained with RBMPS showing the eight areas from the central and peripheral regions used for quantification. **C** Retinal whole-mounts labeled with RBPMS (red) and Tuj1 (green) to visualize RGCs at 7 days post-IR injury. Scale bar: 50 µm. **D** At 7 days post-IR injury, representative HE-stained images of retinal sections from central and peripheral regions. Scale bars: 50 μm. **E** Quantification of the RGC survival rate in both central and peripheral regions of Blank, IR + Vehicle, and IR + EMPA retinas (*n* = 5–6). **F** Measurements of central and peripheral retinal IPL thickness among groups (*n* = 6)

The mice were killed on day 1, day 3, and day 7 after retinal IR injury, and their eyeballs or retinas were harvested for further analyses (Fig. 1A).

### Cell culture and treatment

The BV2 cell line was obtained from Zhong Qiao Xin Zhou Biotechnology Company (Shanghai, China). The cells were cultured in Dulbecco’s modified Eagle’s medium (DMEM, Thermo Fisher Scientific, USA) supplemented with 10% (vol/vol) fetal bovine serum (FBS, Thermo Fisher Scientific, USA), and 1% penicillin/streptomycin (PS, Thermo Fisher Scientific, USA) in a sterile humidified environment at 37 °C and 5% CO2.

Lipopolysaccharides (LPS) can induce a robust mitochondrial fission response and upregulate proinflammatory mediators in BV2 cells [35, 36]. To induce mitochondrial dysfunction and establish an inflammatory model, BV2 cells were exposed to 1 µg/ml of LPS (Sigma-Aldrich, China) for 24 h, with or without co-treatment with EMPA (50 μM).

### Immunofluorescence (IF) staining and quantification

**Fig. 2:**
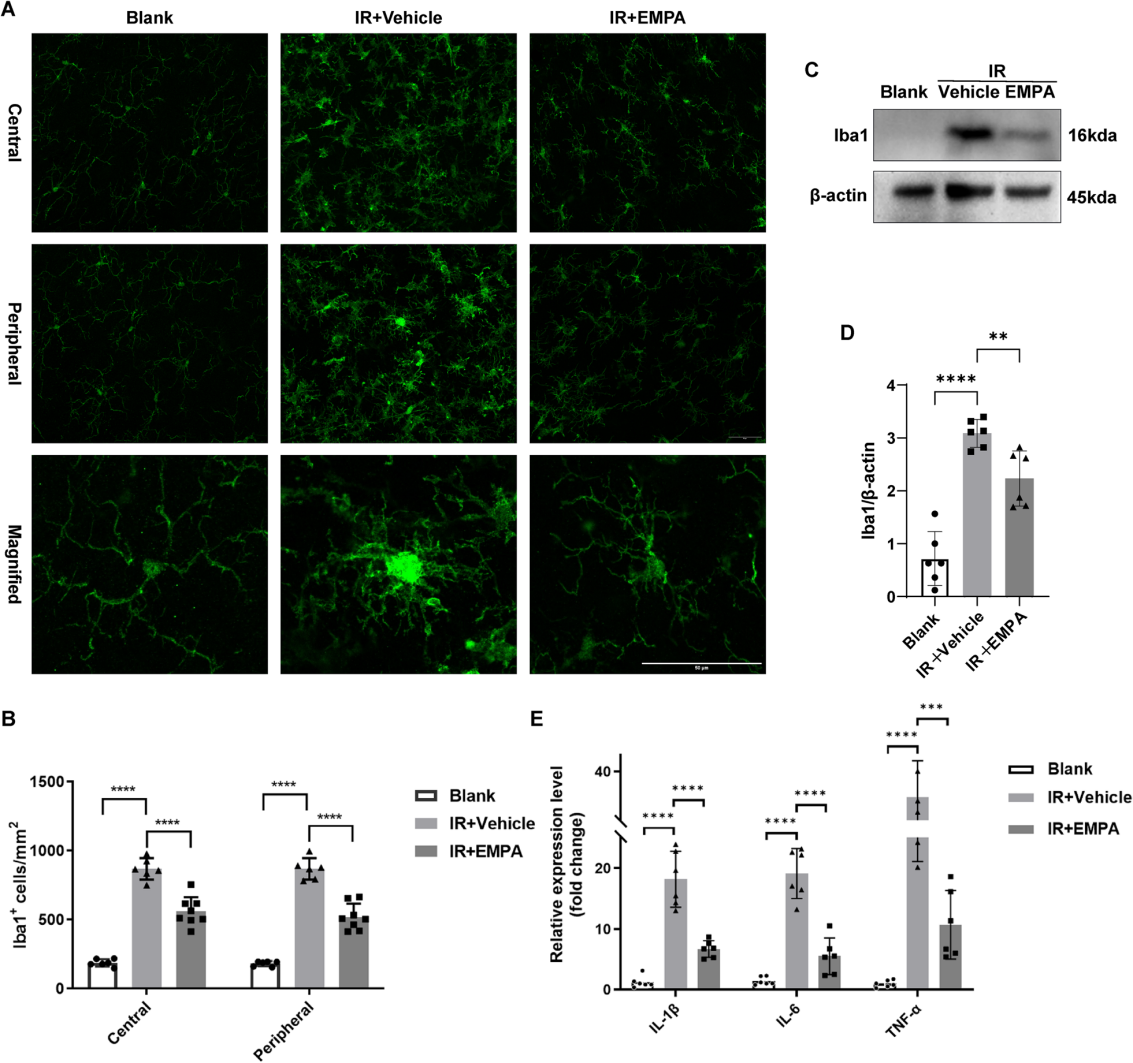
EMPA inhibits microglia activation and neuroinflammation resulting from IR injury. **A** Immunostaining of whole-mount retinas of Blank, IR + Vehicle, and IR + EMPA groups with the Iba 1 antibody (green) at 3 days post-IR injury. Magnified images to show the morphology changes of microglia. Scale bar: 50 μm. **B** Statistic analysis of Iba 1^+^ microglia density in central and peripheral regions among groups (*n* = 6–8). **C** The protein levels of Iba 1 were evaluated by Western blotting. β-actin served as the internal protein control. **D** Relative densitometry quantitation of Iba 1 expression in 3 groups (*n* = 6). **E** 1 day after IR injury, the mRNA expression levels of IL-1β, IL-6, and TNF-α of retinas were measured with qRT-PCR (*n* = 6)

The mice were first transcardially perfused with NS solution, followed by 4% paraformaldehyde (PFA). Then, the eyeballs were enucleated and fixed in 4% PFA for 2 h. For whole-mount retina labeling, the retinas were carefully harvested and permeabilized in 0.5% Triton X-100. After blocking, the collected retinas were incubated with primary antibodies overnight at 4 °C. The next day, the samples were incubated with species-compatible secondary antibodies for 2 h at room temperature (RT). The retinas were then tiled on glass slides and cut into a four-leaf clover shape. We used a Leica DMi8 (Leica Microsystems, Wetzlar Deutschland, Germany) or Zeiss LSM980 confocal microscope (Carl Zeiss, Jena, Thuringia, Germany) to capture both the central and peripheral regions of each quadrant, which were approximately 1 mm and 2 mm away from the optic disc, respectively (Fig. 2B). The average number of RNA binding protein, mRNA processing factor (RBMPS) or Iba1-positive cells of both central and peripheral areas was counted as the mean density of all retinas per group.

For cell labeling, cells grown on matrigel-precoated glass coverslips were fixed in 4% PFA for 30 min at RT. The cells were incubated in appropriate concentrations of serum and Triton X-100 for 1 h. After incubation with primary antibodies overnight, the samples were washed and incubated with the appropriate secondary antibody for 1 h. Cell nuclei were stained by 4,6-diamidino-2-phenylindole (DAPI, Bioss, China) for 10 min. Images were captured using a Leica DMi8 microscope (Leica Microsystems, Wetzlar Deutschland, Germany) and analyzed using ImageJ (https://imagej.nih.gov/ij/). The sources and dilutions of all antibodies for IF are listed in Additional file 1: Table S1.

### Hematoxylin and eosin (HE) staining

The eyeballs were enucleated on the 7th day after the operation and fixed in FAS Fixator (Servicebio, China) for 24 h at RT. Subsequently, the samples were embedded in paraffin and sectioned into 10-μm-thick sections stained with HE solution. Images of the stained sections were obtained with a Leica DM6B microscope. The thickness of inner plexiform layer (IPL) of four cross-sectional areas (500 and 1000 μm from the optic nerve head of both nasal and temporary retina) was measured using ImageJ.

### Western blotting (WB)

Total protein was extracted from retinas and cultured cells using the Whole Cell Protein Extraction Kit (KeyGEN, China). The protein concentration was quantified using the BCA kit (Beyotime, China). Equal amounts of proteins (20-40 μg) were separated on 4–12% gradient polyacrylamide gels at 120 V and transferred to polyvinylidene fluoride (PVDF) membranes at 70 V. The membranes were blocked with 5% skim milk for 1 h at RT and then incubated with primary antibodies overnight at 4℃. On the following day, the membranes were washed three times with Tris-buffered saline containing Tween 20 (TBST) and incubated with horseradish peroxidase-conjugated secondary antibodies. After washing, the protein bands were finally visualized using the enhanced chemiluminescent (ECL, NCM, China) and quantified using ImageJ. The expression levels of target proteins were normalized to β-actin or glyceraldehyde-3-phosphate dehydrogenase (GAPDH) obtained from the same sample. Primary antibodies for Western blotting are listed in Additional file 1: Table S1.

### Reverse transcription (RT) and quantitative real-time PCR (qRT-PCR)

The total RNA of retinas and cells were extracted using the RNA-Quick Purification Kit (ESscience, China). RNA purity and concentration were determined using a NanoDrop spectrophotometer (ND-1000, NanoDrop Technologies, USA). Subsequently, the extracted RNA was transcribed into cDNA using Evo M-MLV RT reagent kit (Accurate Biology, China). qRT-PCR was performed using the SYBR qPCR Master Mix (Accurate Biology, Changsha, China) in the Light Cycler 480 Real-Time PCR System (Roche Molecular Systems, Inc., SUR). Relative mRNA expression levels were normalized to the expression of GAPDH gene in the same samples and calculated using the comparative cycle threshold method (2-ΔΔCt method). The primers targeting corresponding mouse genes are listed in Additional file 1: Table S2.

### Cell viability assay

CCK-8 assay kit (Beyotime, China) was used to assess cell viability in BV2 cells. Briefly, cells were seeded into 96-well plates and treated with the indicated concentration of EMPA for 24 h. Next, 10 μl of CCK-8 buffer was added to each well and incubated at 37 ℃ for 1 h. Absorbance at 450 nm in each well was then measured via a multimode reader.

### Mitochondrial membrane potential (MMP) measurement and mitochondrial reactive oxygen species (mtROS) staining

Mitochondrial membrane potential was assessed using a tetramethylrhodamine, methyl ester (TMRM) probe (Thermo Fisher Scientific, USA). Mito-SOX Red (Thermo Fisher Scientific, USA) was used to detect mtROS, and Hoechst (Beyotime, China) was used to stain the nuclei of living cells. Briefly, target cells were, respectively, seeded in 24-well plates or 35-mm confocal dishes (Biosharp, China), treated accordingly, and stained with TMRM or Mito-SOX staining solution at 37 °C for 30 min. Cells were imaged under a Leica DMi8 (Leica Microsystems, Wetzlar Deutschland, Germany) or Zeiss LSM980 confocal microscope (Carl Zeiss, Jena, Thuringia, Germany) and analyzed using ImageJ.

### Small interfering RNA (siRNA) transfection

Cells were seeded in 6-well plates and grown to 30% confluency before transfection. Mitofusin 1 (Mfn1), Optic atrophy 1 (Opa1), and negative control (NC) siRNA were, respectively, transfected into cells using Lipofectamine RNAiMAX reagent (Thermo Fisher, USA) according to the manufacturer’s instruction. The transfection complexes were prepared with 7.5 μl Lipofectamine RNAiMAX and 3 μl siRNA (30 nm) in 250 μl OptiMEM (Thermo Fisher, USA) per well. The transfected cells were incubated at 37 °C for 24 h, followed by the aforementioned treatments. SiRNAs (RiboBio, China) with the following sequences were used to silence Mfn1 and Opa1 expression: si-Mfn1 #1: GCACACTATCAGAGCTAAA; si-Mfn1 #2: CAAGAAATGGCCACTACTT; si-Mfn1 #3: ACTCAAAGCTCTTAAGAAA; si-Opa1 #1: GCTTACATGCAGAATCCTA; si-Opa1 #2: GCTAAAGACAAGCATGCTA; si-Opa1 #3: CCATGTGGCCTTGTTTAAA.

### Single-cell RNA sequencing analysis

Single-cell RNA sequencing libraries were constructed using the 10 × Genomics kit. CellRanger (version 7.0.0) count commands were applied to demultiplex and barcode the sequences. Downstream analysis was processed in the R Seurat package (version 4.1.1)[37] unless otherwise specified. After stringent quality control (mitochondrial gene ratio less than 20%, detected genes more than 200 and less than 4000), 55,491 cells (Blank, 9282 cells; IR, 26,255 cells; and EMPA-treated IR mice, 19,954 cells) were retained for subsequent analysis. We used the R package harmony (version 0.1.0) to remove the batch effect of sequence data from different groups. The "FindMarkers" function of the Seurat package was used to perform differential expression analysis. Genes with | Log2 (Fold Change) |> 0.25 and *P* value < 0.05 were defined as differentially expressed genes (DEGs).

### Gene functional analysis

DEGs were mapped to functional Gene Ontology (GO) terms using webtool Metascape [38]. Several representative GO terms or pathways were visualized in the R package ggplot2 (version 3.3.6).

### Gene set score analysis

An inflammatory gene set was retrieved from GO term inflammatory response (GO:0006954). Inflammatory gene score was conducted using the Seurat function AddModuleScore. Statistical analysis was conducted with the two-sided Wilcoxon rank sum test.

### Gene set enrichment analysis (GSEA)

Gene sets related to inflammatory response and neuron death were downloaded from MSigDb (https://www.gsea-msigdb.org/gsea/index.jsp). GSEA of IR-DEGs and EMPA-DEGs mapped to corresponding gene sets was performed using R package GSEABase (version 1.56.0).

### Statistic analysis

Statistical analysis was performed using GraphPad Prism 9 software. The data were expressed as mean ± standard error of the mean (SEM). One‐way analysis of variance followed by Dunnett’s multivariate analysis was used to determine the significance of differences among groups. A *P*-value < 0.05 was considered statistically significant. Significance scores are ns for not significant, * for *P* < 0.05, ** for *P* < 0.01, *** for* P* < 0.001, and **** *P* < 0.0001.

## Results

### EMPA performs neuroprotective effects in the retinal IR model

To evaluate the neuroprotective effects of EMPA in retinal IR injury, we conducted an intravitreal injection of EMPA immediately after completing the anterior chamber perfusion (Fig. 1A). After a 7-day reperfusion period, immunostaining of the whole-mount retinas with RBPMS and TUJ1 antibodies revealed a significant reduction in the number of RGCs in both central regions and peripheral regions. However, EMPA administration led to enhanced RGC survival in IR-injured retinas (Fig. 1C, E). Additionally, using HE staining, we measured and compared the thickness of the retinal IPL layer in all experimental groups. At 7 days post-IR injury, we observed a significant decline of the IPL layer thickness in the untreated IR group, which was partially reversed by EMPA treatment (Fig. 1D,F). These findings demonstrated the potential of EMPA in preventing IR-induced retinal morphological damage and RGC loss.

### EMPA inhibits microglial activation and neuroinflammation resulting from IR injury

As previously mentioned, neuroinflammation mediated by microglia can result in RGC degeneration and significantly contribute to the pathogenesis of IR injury. Therefore, we further explored whether EMPA could exert neuroprotective effects by mitigating microglial activation and reducing inflammation. To evaluate the morphology and distribution of microglial cells in retinas, we performed immunofluorescent labeling of whole-mount retinas using Iba1. Our analysis showed that by 3 days after IR injury, EMPA-treated retinas exhibited a 30–50% reduction of microglia compared to untreated ones (Fig. 2A, B). Furthermore, WB analysis demonstrated significantly lower levels of Iba1 protein in the IR + EMPA group compared to the IR + Vehicle group, consistent with the findings of whole-mount retina labeling (Fig. 2C, D). Microglial activation is characterized by a transition from a resting ramified morphology to an amoeboid shape [39]. In the Blank group, microglial cells had small somas and highly ramified branches, whereas in the untreated IR group, the somas appeared enlarged, and the branches exhibited partial retraction, indicating microglial activation. The microglia in the EMPA-treated group displayed a morphology more similar to inactivated microglia, suggesting that intravitreal injection of EMPA exerted an inhibitory effect on microglial activation (Fig. 2A).

Previous studies have established a strong correlation between microglial activation and increased inflammation [40]. Based on this premise, we assessed the mRNA levels of interleukin‐1β (IL-1β), interleukin‐6 (IL-6), and tumor necrosis factor-α (TNF-α), which are the most common pro-inflammatory cytokines. We observed a significant upregulation at 1 day after IR injury, while their expression was significantly downregulated upon EMPA administration.

### EMPA alleviates inflammation and neuron death in the IR retina as assessed by single-cell analysis

**Fig. 3:**
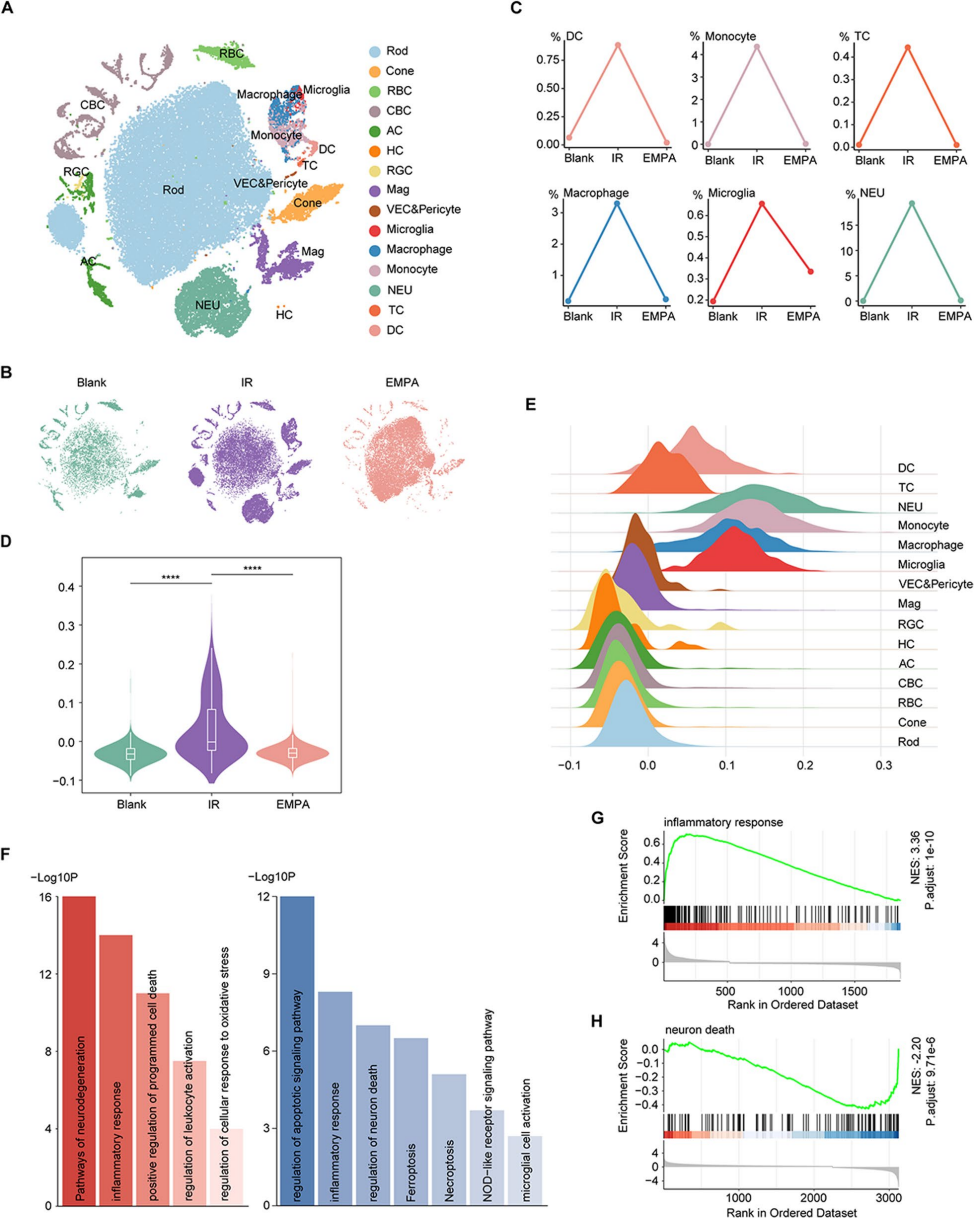
EMPA relieves inflammation and neuron death in IR retinas as assessed by single-cell analysis. **A** A total of 55,491 cells (Blank, 9282 cells; IR, 26,255 cells; and EMPA-treated IR mice, 19,954 cells) were retained for downstream analysis. t-SNE plot showing the distribution of different retinal cell types. **B** t-SNE plot showing the distribution of retinal cells from three groups. **C** Line charts showing the ratio alterations of different clusters of immune cells in all retinal cells. **D** Violin plot showing the inflammatory scores of three groups. For the box plot within each violin plot, the middle line of the box within the violin indicates the median value and the box ranges from the 25th to 75th percentile. **E** Ridge plots showing the inflammatory scores of different cell types. **F** Bar plots showing the GO terms enriched for the upregulated IR-DEGs (left) and downregulated EMPA-DEGs (right) of overall retinal cells. The color from light to dark indicates the statistical significance value from low to high. **G** GSEA of IR-DEGs mapping to GO pathway inflammatory response. **H** GSEA of EMPA-DEGs mapping to GO pathway neuron death

Single-cell RNA sequencing (scRNA-seq) provides unprecedented resolution of information from multiple tissues and organs, allowing the investigation of molecular cascades in individual cell types under biological and pathological conditions. Hence, using single-cell RNA analysis, we constructed a comprehensive atlas of Blank, IR, and EMPA-treated IR mice. Based on canonical marker genes, we identified 15 major cell populations: rod photoreceptor (Rod), cone photoreceptor (Cone), rod bipolar cell (RBC), cone bipolar cell (CBC), amacrine cell (AC), horizontal cell (HC), retinal ganglion cell (RGC), macroglia, vascular endothelial cell (VEC) plus pericyte, microglia, macrophage, monocyte, neutrophil (NEU), T cell (TC) and dendritic cell (DC) (Fig. 3A, Additional file 1: Fig. S1A). Notably large clusters of immune cells, especially myeloid cells, were observed after IR but were significantly reduced following EMPA treatment (Fig. 3B).

To further explore the anti-inflammatory effect of EMPA, we analyzed the exact cell ratio of immune cells (Fig. 3C), which were remarkably increased after IR injury and decreased after EMPA treatment. We also calculated the inflammatory scores based on the expression of inflammatory signatures in individual cells and various cell types. The IR group exhibited elevated inflammatory scores compared to the Blank group, whereas EMPA treatment reversed these scores (Fig. 3D). Moreover, myeloid cells generally presented with higher scores than other immune cells and retinal neurons (Fig. 3E).

We then performed differential expression analysis between different groups, identifying differentially expressed genes (DEGs) between IR and Blank mice (referred to as IR-DEGs) and between EMPA-treated IR mice and IR mice (referred to as EMPA-DEGs) (see Methods for details). After functional annotation of these DEGs, we found that upregulated IR-DEGs mainly corresponded to inflammation and oxidative stress, which were attenuated by EMPA treatment (Fig. 3F). Notably, downregulated EMPA-DEGs were enriched in GO terms related to different types of cell death, suggesting the potential neuroprotective effects of EMPA in promoting neuron survival under IR-induced stress. We observed a decrease in retinal visual function after IR injury, which was subsequently rescued by EMPA treatment (Additional file 1: Fig. S1B). In addition, EMPA could regulate mitochondrial dynamics, as indicated by GO mitochondrion morphogenesis. We further dissected the anti-inflammation and anti-death effect of EMPA by mapping IR-DEGs and EMPA-DEGs to gene sets associated with inflammatory response and neuron death (see Methods for details). Gene set enrichment analysis (GSEA) revealed that EMPA prominently alleviated inflammation and neuron death after IR damage (Fig. 3G, H, Additional file 1: Fig. S1C-D).

### EMPA ameliorates inflammation through the regulation of mitochondria homeostasis in microglia

**Fig. 4:**
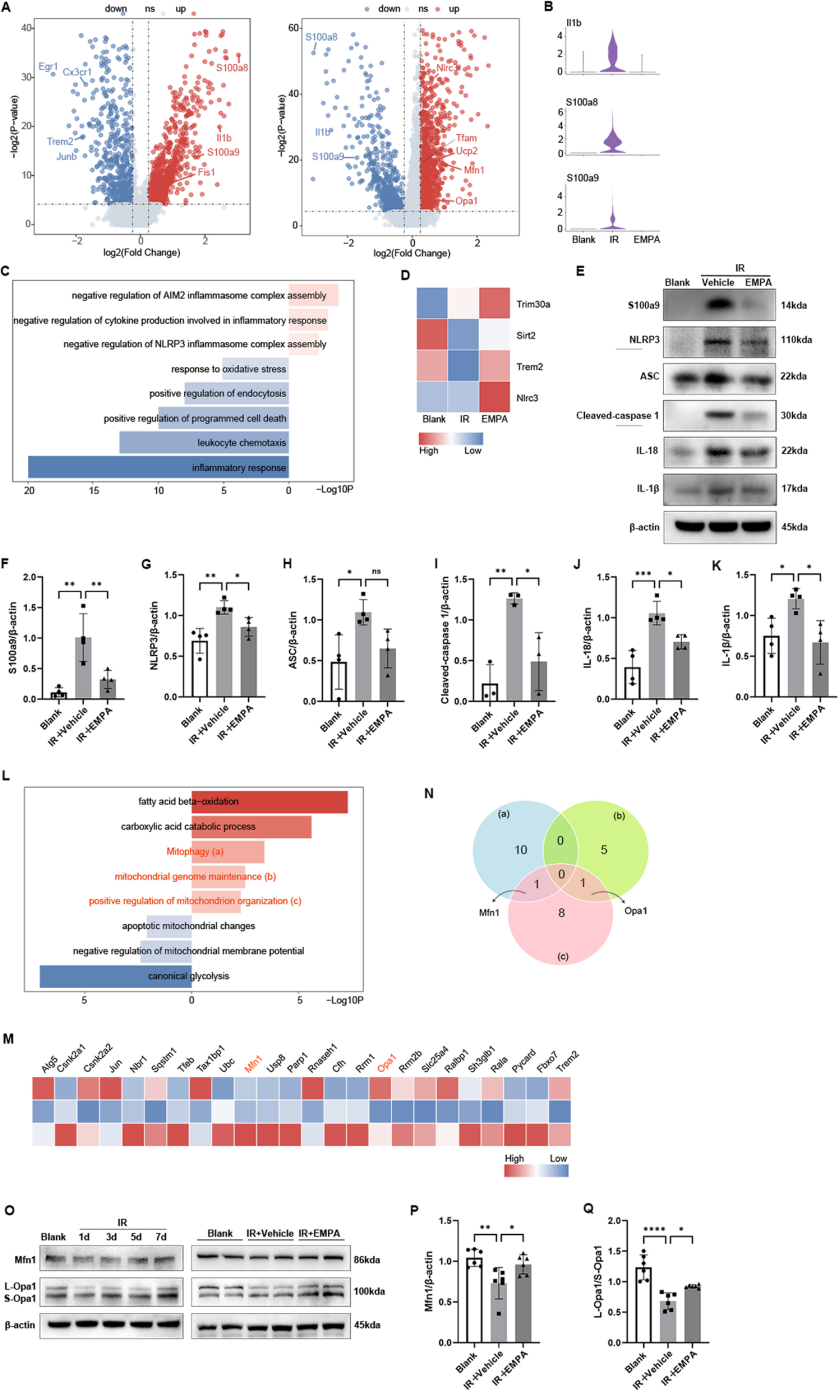
EMPA ameliorates inflammation through the regulation of mitochondria homeostasis in microglia. **A** Volcano plots showing upregulated and downregulated IR-DEGs (left) and EMPA-DEGs (right) of microglia. Red and blue dots indicate upregulated and downregulated DEGs in the corresponding comparison groups, respectively. **B** Violin plots showing the expression of indicated genes among three groups in microglia. **C** Bar plots showing the GO terms related to inflammation enriched for the upregulated (red) and downregulated EMPA-DEGs (blue) of microglia. The color from light to dark indicates the statistical significance value from low to high. **D** Heatmap showing the expression of genes mapped to GO pathways negative regulation of NLRP3 inflammasome complex assembly from three groups in microglia. The color key from blue to red indicates the expression level from low to high. **E** On 3 days post-IR injury, Western blotting analysis was performed to detect the expression of S1009a9 and NLRP3 inflammasome pathway-related proteins among 3 groups. β-Actin served as the internal protein control. **F**–**K** Relative densitometry quantitation of relative protein expression levels (*n* = 3–4). **L** Bar plots showing the GO terms related to mitochondria function enriched for the upregulated (red) and downregulated EMPA-DEGs (blue) of microglia. The color from light to dark indicates the statistical significance value from low to high. **M** Heatmap showing the expression of genes mapped to GO pathways from three groups in microglia. The color key from blue to red indicates the expression level from low to high. **N.** Venn diagrams showing the intersection of genes enriched in upregulated GO terms related to mitochondria function including (a) mitophagy, (b) mitochondrial genome maintenance, (c) positive regulation of mitochondrion organization after EMPA treatment in microglia. **O** Western blotting analysis showed Mfn1 and Opa1 expression changes at different time points after IR injury. **P** On 3 days post-IR injury, Western blotting analysis was performed to detect the expression of Mfn1 and Opa1 among 3 groups. β-Actin served as the internal protein control. **Q**, **R** Relative densitometry quantitation of Mfn1 and Opa1 expression levels (*n* = 6)

Microglia, the resident immune cell of the retina, are crucial players in maintaining retinal homeostasis under normal conditions, as well as mediating inflammatory response in diseases [8]. Hence, we focused on microglia, particularly in comparing IR and EMPA treatment groups in the downstream analysis. As depicted in Additional file 1: Fig. S2A, the distribution of microglia in the EMPA group exhibited greater similarity to that of the Blank group rather than the IR group. Additionally, there was a general downregulation of M1 markers and an opposite trend observed for M2 markers in response to EMPA treatment (Additional file 1: Fig. S2B). These findings serve as evidence that EMPA effectively inhibits the transition of microglia towards a neurotoxic phenotype. Under IR stress, genes involved in the sensing and housekeeping properties of microglia, such as Trem2 and Cx3cr1, were downregulated (Fig. 4A). These genes are important for controlling microglial inflammatory responses. Conversely, the expression of pro-inflammatory genes, including S100a8, S100a9, and Il1b was notably elevated (Fig. 4A, B), which was greatly reversed by EMPA treatment. To further explore the anti-inflammatory effect of EMPA on microglia, we performed pathway analysis of EMPA-DEGs. GO terms related to oxidative stress and leukocyte chemotaxis were decreased, and EMPA also negatively regulated the assembly of inflammasome complex (Fig. 4C). Trim30a, Sirt2, Trem2, and NIrc3 were enriched in the term negative regulation of NLRP3 inflammasome complex assembly (Fig. 4D). The most upregulated gene Nlrc3, a member of negative regulatory NLRs, plays a key role in attenuating the excessive inflammation [41]. Consistent with the scRNA-seq findings, WB analysis indicated that IR injury upregulated the expression of S1009a9 and proteins related to NLRP3 inflammasome pathway, which were partially reversed by EMPA treatment (Fig. 4E–K).

Given the growing evidence of EMPA’s multifaceted effects on mitochondrial functions [29–32], we speculated that EMPA might mitigate neuroinflammation through regulating mitochondria homeostasis. In the context of retinal IR, EMPA-DEGs were significantly enriched in mitochondrial remodeling (Fig. 4L), demonstrating that EMPA inhibited mitochondrial apoptosis and improved mitochondrial membrane potential and mitophagy. EMPA was also involved in regulating metabolic reprogramming. EMPA-DEGs were further enriched in mitochondrial genome maintenance and positive regulation of mitochondrion organization. We calculated the average expression of genes mapped to upregulated GO terms related to mitochondrial function after EMPA treatment, which showed varying degrees of upregulation (Fig. 4M). To identify key players mediating the mitochondria-promoting effect, we performed the Venn analysis of these genes. Notably, Mfn1 and Opa1, which, respectively, participate in mitochondrial fusion of the outer and inner membranes, were the only two genes among the intersection (Fig. 4N). We examined the protein levels of Mfn1 and Opa1 and found that both of them were downregulated on day 1, day 3, and day 5 after retinal IR insult but restored on day 7. As expected, EMPA alleviated IR-induced downregulation of Mfn1 and long Opa1 isoform to short Opa1 isoform (L-Opa1/S-Opa1) ratio (Fig. 4O–Q). Collectively, EMPA treatment increases the expression of mitochondrial fusion-related proteins and improves mitochondrial homeostasis, which might be an important mechanism through which EMPA reduces inflammation in retinal IR models.

### EMPA attenuates LPS-induced cell inflammation and NLRP3 inflammasome activation in BV2 cells

**Fig. 5:**
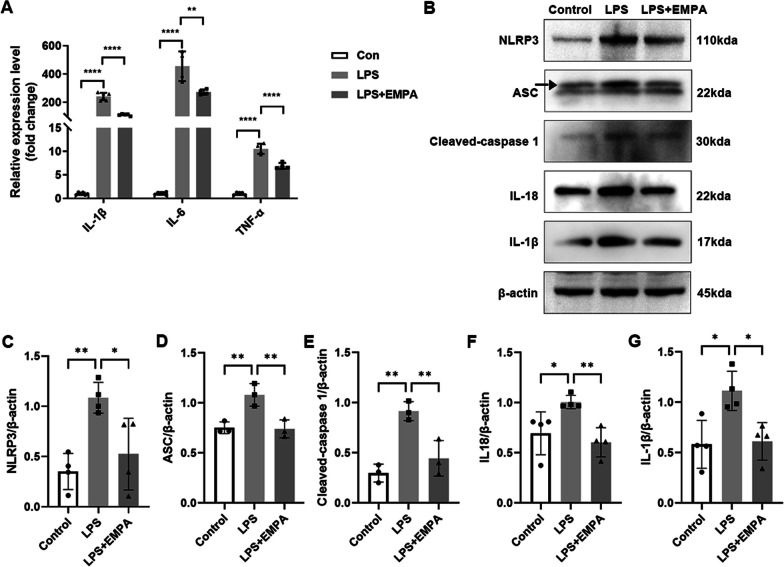
EMPA attenuates LPS-induced inflammation and NLRP3 inflammasome activation in BV2 cells. **A** qRT-PCR analysis of mRNA expression levels of inflammatory factors (IL-1β, IL-6, TNF-α) in control and LPS-stimulated BV2 cells treated with or without EMPA (*n* = 4–6). **B** Western blotting analysis was carried out to detect NLRP3 inflammasome pathway-related proteins. β-Actin served as the internal protein control. **C**–**G** Relative densitometry quantitation of relative protein expression levels in the 3 groups of BV2 cells (*n* = 3–4)

After demonstrating the favorable anti-inflammatory effects of EMPA in the IR model, we subsequently focused on examining the role of EMPA in vitro. As shown in Additional file 1: Fig. S3A, the viability of BV2 cells remained largely unaffected by EMPA within the dosage range of 1–50 μM. To evaluate the anti-inflammatory effects of EMPA in vitro, BV2 cells were subjected to LPS stimulation. IL-1β, IL-6, and TNF-α were selected as representative pro-inflammatory factors. LPS exposure increased the mRNA levels of these pro-inflammatory mediators, which were significantly inhibited by EMPA in BV2 cells (Fig. 5A). In addition, EMPA demonstrated the ability to inhibit the LPS-induced upregulation of NLRP3 inflammasome-related proteins, including NLRP3, apoptosis-associated speck-like protein (ASC), caspase-1 activation, the maturation of IL-18 and IL-1β, as confirmed by WB analysis (Fig. 5B–G).

In Additional file 1: Fig. S2, EMPA was found to suppress the LPS-induced polarization of BV2 cells towards the M1 phenotype (pro-inflammatory) while promoting polarization towards the M2 phenotype (anti-inflammatory). The immunofluorescence intensity of iNOS, a commonly employed marker for detecting the M1 phenotype, was increased following LPS insult but decreased in the presence of EMPA (Additional file 1: Fig. S3B, C). Additionally, qPCR analysis was utilized to examine markers of M1 and M2 phenotypes. The mRNA expression of M1 markers, including CD86, CD32, COX2, and iNOS, was significantly reduced by EMPA treatment compared with the LPS groups, whereas the expression of M2 markers such as CCL-22 and Arg1 was significantly increased (Additional file 1: Fig. S3D, E).

### EMPA reverses LPS-induced mitochondrial dysfunction in BV2 cells

**Fig. 6:**
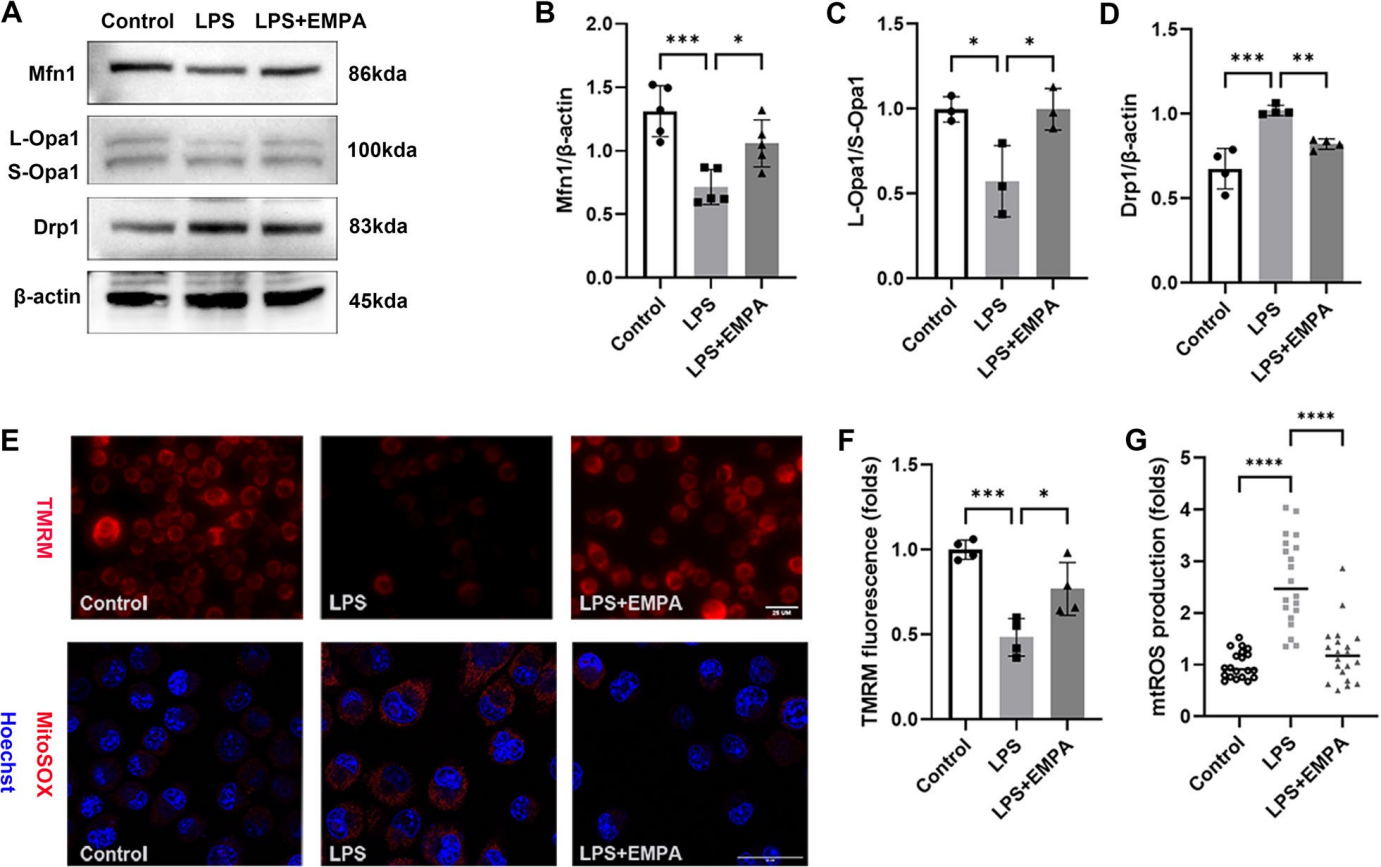
EMPA promotes mitochondrial fusion, and suppresses MMP decline and mtROS production in BV2 cells treated with LPS. **A** Western blotting analysis was used to assess the expression of Mfn1, Opa1, and Drp1 in control and LPS-stimulated BV2 cells treated with or without EMPA. β-Actin served as the internal protein control. **B**–**D** Relative densitometry quantitation of relative protein expression levels in the 3 groups of BV2 cells (*n* = 3–5). **E**–**G** The MMP and mtROS levels in BV2 cells were determined using immunofluorescence analyses. The MMP was determined by the fluorescence intensity of TMRM staining, and statistical analyses were determined by Image J (**F**) (*n* = 3). The mtROS was visualized by MitoSOX staining, and statistical analyses were determined by Image J (**G**) (*n* = 20). Scale bar: 25 µm

Mitochondrial damage plays a pivotal role in innate immunity and the inflammatory response [12, 42]. In light of this, we proceeded to evaluate the protective effects of EMPA on mitochondrial injury in BV2 cells. WB analysis demonstrated that EMPA treatment upregulated Mfn1 and L-Opa1, proteins implicating mitochondrial fusion, and downregulated the mitochondrial fission-related protein Drp1 (Fig. 6A–D). This indicates that EMPA normalized mitochondrial dynamics in LPS-stimulated BV2 cells. The timely removal of fragmented mitochondria via mitophagy is essential for maintaining mitochondrial morphology and function [43]. Therefore, the levels of mitophagy markers were analyzed. Microtubule-associated protein 1 light chain 3 beta (LC3B) is necessary for autophagy membrane formation and P62 interacts with LC3B to facilitate the delivery of damaged mitochondria and other autophagosomes to autolysosomes for degradation [44]. LPS stimulation reduced the expression of LC3B II and elevated P62 accumulation, suggesting impaired mitophagy. In contrast, the LPS + EMPA group showed enhanced LC3B II expression and decreased P62 levels (Additional file 1: Fig. S4).

In the LPS group, increased mitochondrial fission led to the loss of MMP and elevated mtROS levels in BV2 cells. TMRM dye was used to quantify changes in MMP. TMRM dye would accumulate in active mitochondria with intact MMP and exhibit red fluorescence, but the red fluorescence dims or disappears once MMP is lower or lost. We observed that EMPA treatment partially restored MMP and mitigated the production of mtROS (Fig. 6E–G). Generally speaking, LPS-induced damage was characterized by disordered mitochondrial dynamics, inhibited mitophagy, reduced MMP, and increased mtROS. EMPA attenuated these pathological alterations in the mitochondria of BV2 cells.

### Inhibiting mitochondrial fusion abolishes the anti-inflammatory effect of EMPA in BV2 cells

**Fig. 7:**
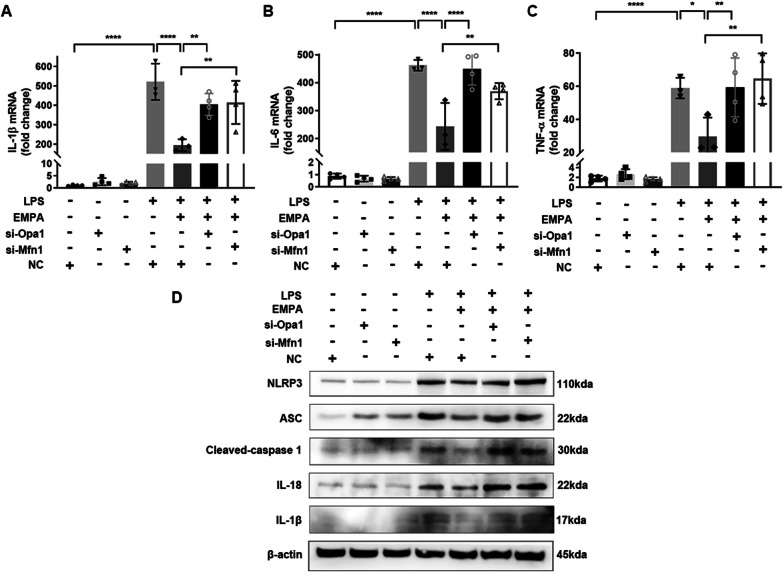
Mfn1 and Opa1 are essential for the anti-inflammatory effect of EMPA in BV2 cells treated with LPS. **A** BV2 cells were transfected with si-Mfn1 or Opa1 and followed with LPS stimulation with or without EMPA for 24 h. mRNA expression levels of inflammatory factors (IL-1β, IL-6, TNF-α) among groups were determined by qRT-PCR analysis (*n* = 3–4). **B** The variances of NLRP3 inflammasome pathway-related proteins were determined by western blotting. β-Actin served as the internal protein control

Subsequently, our investigation aimed to determine whether the effects of EMPA on inflammation were mediated through its influence on mitochondrial dynamics. To achieve this, we transfected siRNAs to block the production of Mfn1 and Opa1 in BV2 cells, and further assessed the efficiency of knockdown (Additional file 1: Fig. S5). The knockdown of Mfn1 and Opa1 effectively abolished the inhibitory role of EMPA on the mRNA levels of pro-inflammatory cytokines (IL-1β, IL-6, and TNF-α) (Fig. 7A–C). Additionally, EMPA was found to partially reverse the upregulation of NLRP3 inflammasome-related proteins in LPS-induced BV2 cells. However, this reversal was not observed with Mfn1 or Opa1 knockdown. These results confirmed that EMPA inhibited inflammation by enhancing the expression of Mfn1 and Opa1, thus restoring mitochondrial dynamics.

## Discussion

**Fig. 8:**
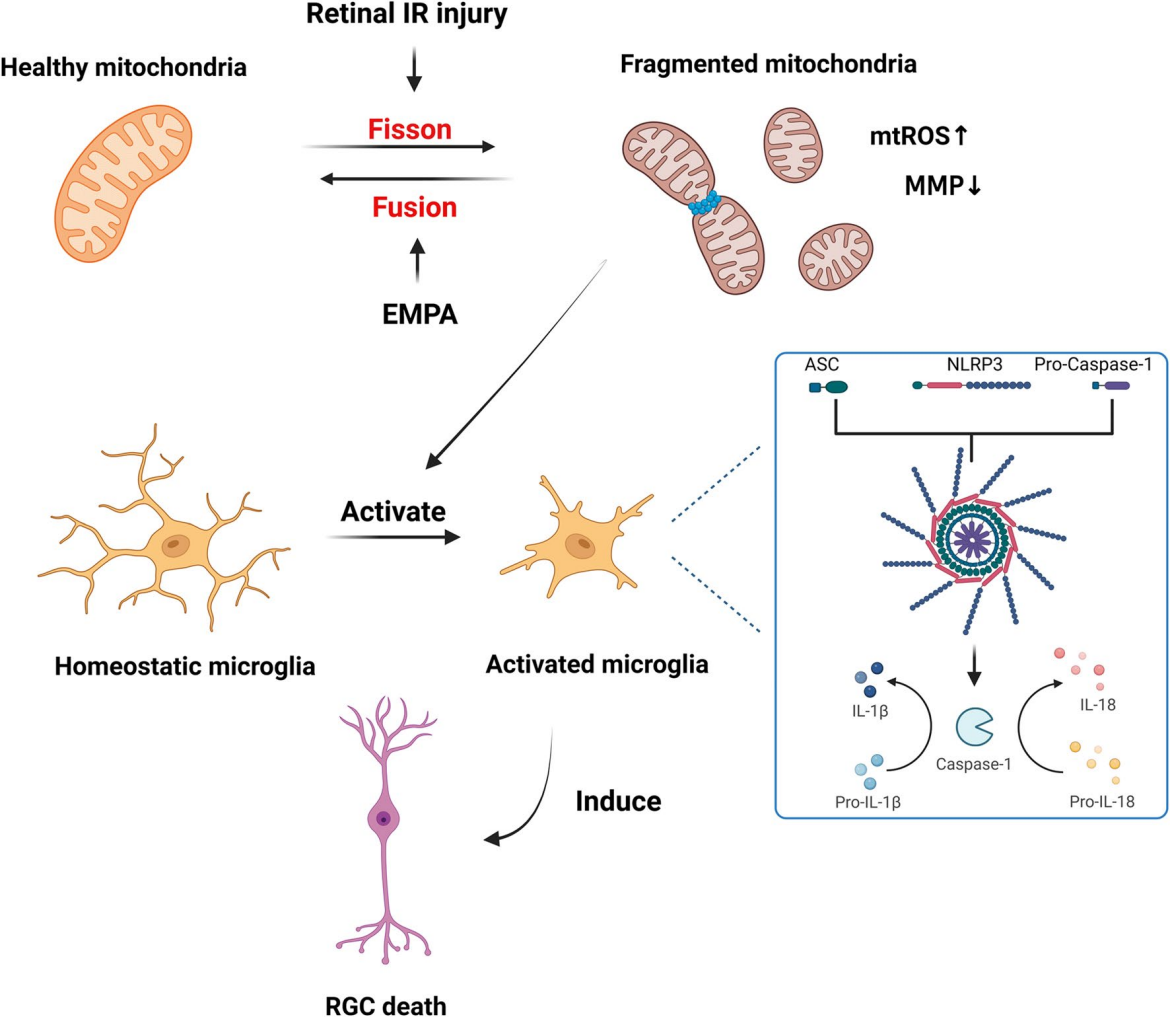
Hypothesized schematic presentation of the protective mechanism of EMPA against retinal IR injury

In the current study, we first investigated the potential therapeutic effects of intraocular EMPA administration in retinal IR injury. We found that EMPA protected against retinal structure disruptions and neuroinflammation caused by IR injury. ScRNA-seq analysis demonstrated that EMPA downregulated microglia-mediated inflammation and the assembly of NLRP3 inflammasome complex by mitigating mitochondrial dysfunction, especially through rebalancing mitochondrial dynamics during retinal IR injury. In vitro studies also confirmed that EMPA alleviated inflammatory responses and NLRP3 inflammasome activation in BV2 cells. However, its anti-inflammatory effect was abolished by Mfn1 or Opa1 deficiency. We revealed that intraocular administration of EMPA during IR injury protected against the loss of Mfn1 and Opa1, improved mitochondrial fusion, attenuated the NLPR3 inflammasome response, and ultimately improved RGC survival. A schematic representation of these findings is presented in Fig. 8.

Retinal IR insult is an established experimental model that mimics the pathological process of retinal neurodegenerative diseases, such as diabetic retinopathy and glaucoma. Retinal IR injury could induce irreversible RGC loss along with the thinning of retinal inner layers [3, 9–11], in line with our current findings. Previous studies have found that EMPA administration exerts protective effects in cerebral, cardiovascular, and renal IR models [31, 45, 46]. These studies suggest that EMPA could activate specific signaling cascades to attenuate oxidative stress, alleviate inflammation, and induce pro-survival programs in various cell types. Consistent with these findings, our results demonstrate that intravitreal injection of EMPA effectively prevents the loss of retina tissues and RGC induced by retinal IR injury.

The crosstalk between microglial activation and neuronal loss plays an imperative role in the progression of neurodegenerative diseases [47]. Under pathological conditions, including IR injury, overactivated microglia can potentiate RGC death by producing proinflammatory neurotoxic cytokines and engaging in phagocytosis of living neurons [8, 48]. In our study, EMPA suppressed the proliferation and activation of microglia, reducing the accumulation of proinflammatory cytokines both in vivo and in vitro. ScRNA-seq analysis also confirmed the downregulation of immune cell ratio and inflammatory response after EMPA treatment, along with the relief of cell death.

Further investigation of the microglial subset revealed that EMPA decreased the levels of S100a9 and inhibited the assembly of the NLRP3 inflammasome complex in IR-injured mice. S100a9 belongs to the DAMPs family and plays a vital role in mediating inflammatory reactions by stimulating leukocyte recruitment and cytokine secretion [49]. NLRs are pattern recognition receptors (PRPs) widely distributed in microglia to detect pathogen-associated molecular patterns (PAMPs) or DAMPs. Among the NLR family members, the NLRP3 inflammasome is highly expressed in microglia and is especially relevant to neurotoxicity [49]. Activation of NLRs triggers the assembly of an NLRP3–ASC–caspase-1 complex, known as the inflammasome, which then cleaves pro-interleukins (IL-1β and IL-18) into their active forms. Our previous study indicated that NLRP3 inflammasomes activation was involved in retinal IR injury, and inhibiting NLRP3 could protect the retina against neuronal damage [9, 10, 33].

Mitochondria, as the central hub of the immune system, could modulate the development and function of immune cells [42]. While most studies on neuroinflammation regulation focused on inhibiting certain inflammatory pathways and cytokine activity, recent research highlighted the importance of restoring mitochondrial health as a potent immunomodulatory target [6, 12, 15]. Mitochondria are dynamic organelles that can shift between fused and fragmented morphologies to adapt to environmental changes and metabolic demands [43]. Mitochondrial fission drives the metabolic reprogramming from oxidative phosphorylation to glycolysis and further promotes inflammation [50, 51]. In addition, fragmented mitochondria can result in the overproduction of mtROS, impaired MMP, and the release of mtDAMPs, such as mtDNA [52, 53]. Fragmented and dysfunctional microglial mitochondria have been shown to propagate inflammatory neurodegeneration [18]. Key proteins involved in mitochondrial dynamics include Mfn1/2, Opa1, and Drp1. Mfn1/2 and Opa1 are key regulators of the fusion of mitochondrial outer and inner membranes, while Drp1 mediates mitochondrial fission [17]. S-Opa1 is generated by the cleavage of L-Opa1 in the transmembrane domain. The balanced proteolytic cleavage of Opa1 promotes efficient fusion, whereas excessive accumulation of S-Opa1 may accelerate fission, leading to mitochondrial fragmentation and dysfunction [54]. Rebalancing mitochondrial fusion and fission by overexpression or deletion of these genes and application of specific drugs such as Mdivi-1 was proven to suppress NLRP3 inflammasome and inflammatory responses [55–57]. All the above findings demonstrate the role of abnormal mitochondrial dynamics in driving neuroinflammation and suggest that targeting mitochondrial fission blockade represents a potential strategy for treating various neuronal disorders [19]. Consistent with former works [58–60], we found decreased levels of Mfn1 and a lower ratio of L-Opa1 relative to S-Opa1 in IR-injured retinas, indicating increased mitochondrial fragmentation. EMPA administration repressed mitochondrial fission by restoring Mfn1 and L-Opa1 levels after IR injury, thereby preserving mitochondrial function. In BV2 cells, EMPA treatment upregulated Mfn1 and Opa1 while downregulating Drp1, leading to restored mitochondrial morphologies, reduced mitochondrial oxidative stress, and stabilized MMP. Furthermore, the inhibition of mitochondrial fusion by siRNA blunted the inhibitory effect of EMPA on proinflammatory cytokine release and NLRP3 inflammasome activation in LPS-treated BV2 cells, suggesting that EMPA inhibits neuroinflammation in a mitochondrial fusion-dependent manner.

However, several limitations exist in the present study. Firstly, while Mfn1 and Opa1 have been identified as the upstream mediators of NLRP3 inflammasome and neuroinflammation, it remains unclear whether inflammation has any feedback effects on mitochondrial dynamics in retinal IR injury. Further investigation is needed to fully understand the intricate relationships between mitochondrial fusion/fission and neuroinflammation, particularly in the presence of EMPA. Secondly, the precise mechanisms by which EMPA interacts with Mfn1 and Opa1 are not yet fully understood. Additional research is required to elucidate the specific molecular interactions and signaling pathways involved. Furthermore, additional studies are required to elucidate the interaction of microglia, astrocytes, oligodendrocytes and RGCs within this context.

In summary, the current findings indicate that the effects of EMPA on retinal IR injury are associated with rebalancing mitochondrial fusion and fission, which subsequently leads to the inactivation of NLRP3 inflammasome and the inhibition of neuroinflammation. Our study enhances the current understanding of the interplay between mitochondrial dysfunction and neuroinflammation in the development of retinal IR injury. Additionally, our findings offer a potential novel target for the prevention and treatment of neurodegenerative diseases associated with IR, such as glaucoma and diabetic retinopathy.

### Supplementary Information

**Additional file 1: Fig. S1.** EMPA relieves inflammation and neuron death in the IR retina. **A.** Dot plot showing markers genes (rows) that uniquely mark different cell types in mouse retina (columns). The size of the dot indicates the percentage of cells expressing the gene, and the color represents the average expression level of the gene in the indicated cell types. **B.** Bar plots showing the GO terms enriched for the downregulated IR-DEGs (left) and upregulated EMPA-DEGs (right) of overall retinal cells. The color from light to dark indicates the statistical significance value from low to high. **C.** GSEA of EMPA-DEGs mapping to GO pathway neuron death. **D.** GSEA of IR-DEGs mapping to GO pathway neuron death. **Fig. S2.** EMPA suppresses microglial activation in the IR retina. **A.** t-SNE plot showing the distribution of microglia from three groups. B. Heatmap showing the expression of M1 and M2 markers from three groups in microglia. The color key from blue to red indicates the expression level from low to high. **Fig. S3.** EMPA modulates LPS-induced polarization in BV2 cells treated with LPS. **A.** BV2 cells were treated with indicated concentrations of EMPA. Cell viability was measured by CCK-8 assay. **B.** Representative immunofluorescence images of INOS in control and LPS-stimulated BV2 cells treated with or without EMPA. Scale bar: 50 µm. **C.** The quantity of the intensity of INOS immunofluorescence in BV2 cells (*n* = 3). **D-E.** qRT-PCR analysis of mRNA expression levels of M1 markers (CD68, CD32, COX2, INOS) and M2 markers (CCL-22, Arg1) (*n* = 4–6). **Fig. S4.** EMPA activates mitophagy in BV2 cells treated with LPS. **A.** The levels of mitophagy-related proteins (LC3B and P62) were analyzed through Western blots. β-actin served as the internal protein control. **B.** Relative densitometry quantitation of relative protein expression levels in the 3 groups of BV2 cells (*n* = 3). **Fig. S5.** Knockdown efficiency of Opa1 (**A**) or Mfn1 (**B**) si RNA. **Table S1.** Primary antibodies and dilutions. **Table S2.** qRT-PCR primer sequences.

## Data Availability

The data are available from the corresponding author on reasonable request. The scRNA-seq data are deposited in the Genome Sequence Archive (GSA, https://ngdc.cncb.ac.cn/gsa/) under the GSA Accession No. CRA011537.

## Abbreviations

Mfn1: Mitofusin 1
Opa1: Optic atrophy 1
IR: Ischemia and reperfusion
EMPA: Empagliflozin
RGC: Retinal ganglion cell
scRNA-seq: Single-cell RNA sequencing
NLRP3: Nucleotide-binding domain and leucine-rich repeat containing protein 3
LPS: Lipopolysaccharide
mtROS: Mitochondrial reactive oxygen species
MMP: Mitochondrial membrane potential
IOP: Intraocular pressure
mtDAMPs: Mitochondrial damage related model molecules
mtDNA: Mitochondrial DNA
qRT-PCR: Quantitative real-time PCR
IL‐1β: Interleukin‐1β
IL-6: Interleukin-6
TNF-α: Tumor necrosis factor-α
ASC: Apoptosis-associated speck-like protein containing CARD
IL-18: Interleukin-18
Drp1: Dynamin-related protein 1
RBPMS: RNA binding protein, mRNA processing factor
Tuj1: Tubulin-βIII
HE: Hematoxylin and eosin
IPL: Inner plexiform layer
t-SNE: T-distributed Stochastic Neighbor Embedding
GO: Gene Ontology
DEGs: Differentially expressed genes
GSEA: Gene Set Enrichment Analysis
CCK-8: Cell Counting Kit-8
LC3B: Microtubule-associated protein 1 light chain 3 beta
