# Supplementary material for: Empagliflozin targets Mfn1 and Opa1 to attenuate microglia-mediated neuroinflammation in retinal ischemia and reperfusion injury
Source: J Neuroinflammation. 2023 Dec 12;20:296. doi: 10.1186/s12974-023-02982-9 (PMC10714482; doi:10.1186/s12974-023-02982-9)
Supplement: Supplementary file 1 — Additional file 1: Fig. S1. EMPA relieves inflammation and neuron death in the IR retina. A. Dot plot showing markers genes (rows) that uniquely mark different cell types in mouse retina (columns). The size of the dot indicates the percentage of cells expressing the gene, and the color represents the average expression level of the gene in the indicated cell types. B. Bar plots showing the GO terms enriched for the downregulated IR-DEGs (left) and upregulated EMPA-DEGs (right) of overall retinal cells. The color from light to dark indicates the statistical significance value from low to high. C. GSEA of EMPA-DEGs mapping to GO pathway neuron death. D. GSEA of IR-DEGs mapping to GO pathway neuron death. Fig. S2. EMPA suppresses microglial activation in the IR retina. A. t-SNE plot showing the distribution of microglia from three groups. B. Heatmap showing the expression of M1 and M2 markers from three groups in microglia. The color key from blue to red indicates the expression level from low to high. Fig. S3. EMPA modulates LPS-induced polarization in BV2 cells treated with LPS. A. BV2 cells were treated with indicated concentrations of EMPA. Cell viability was measured by CCK-8 assay. B. Representative immunofluorescence images of INOS in control and LPS-stimulated BV2 cells treated with or without EMPA. Scale bar: 50 µm. C. The quantity of the intensity of INOS immunofluorescence in BV2 cells (n = 3). D-E. qRT-PCR analysis of mRNA expression levels of M1 markers (CD68, CD32, COX2, INOS) and M2 markers (CCL-22, Arg1) (n = 4–6). Fig. S4. EMPA activates mitophagy in BV2 cells treated with LPS. A. The levels of mitophagy-related proteins (LC3B and P62) were analyzed through Western blots. β-actin served as the internal protein control. B. Relative densitometry quantitation of relative protein expression levels in the 3 groups of BV2 cells (n = 3). Fig. S5. Knockdown efficiency of Opa1 (A) or Mfn1 (B) si RNA. Table S1. Primary antibodies and dilutions. Table S2. qRT-PCR [file 12974_2023_2982_MOESM1_ESM.docx]

**
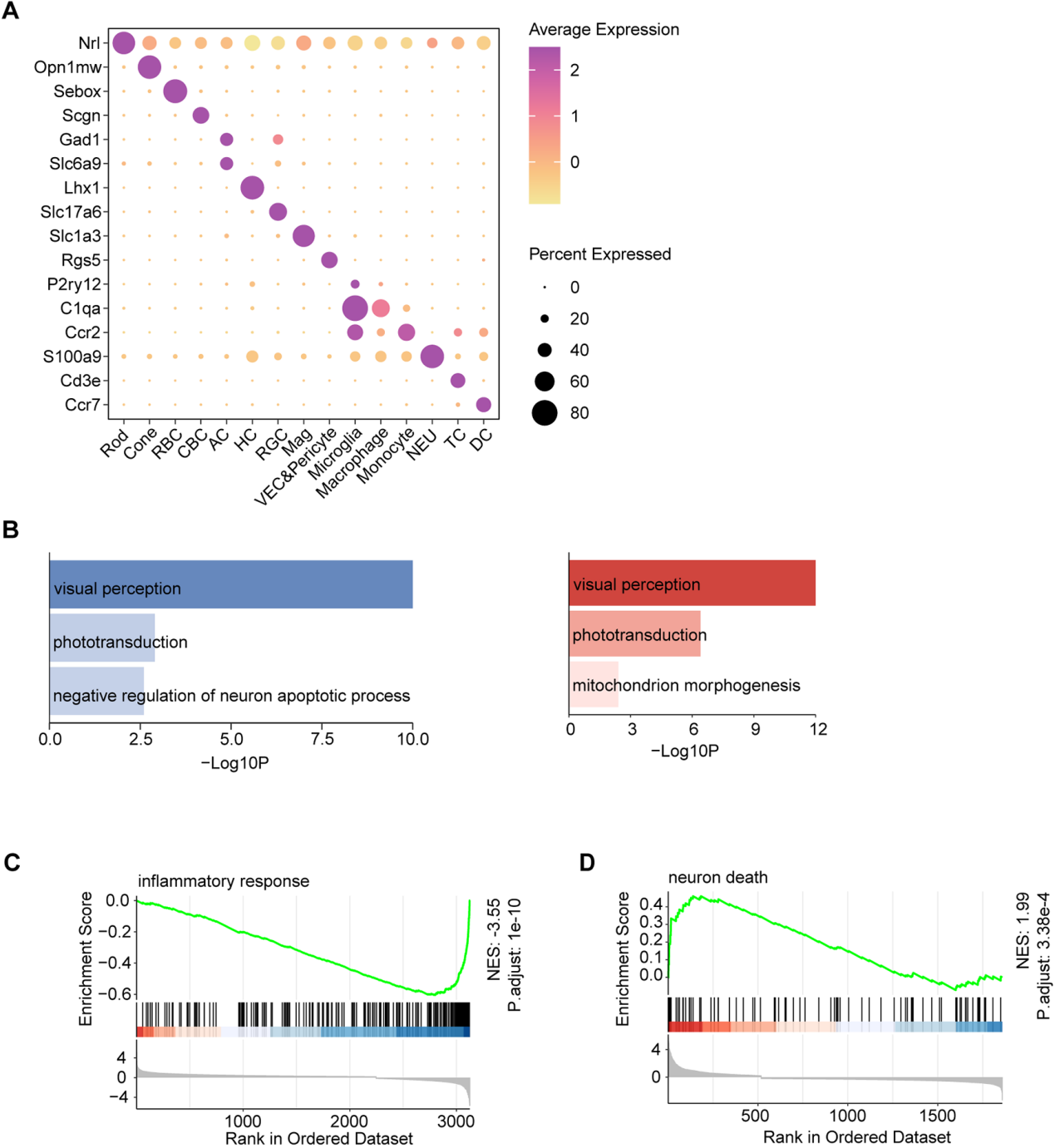
**

**Fig. S1. EMPA relieves inflammation and neuron death in the IR retina**

**A.** Dot plot showing markers genes (rows) that uniquely mark different cell types in mouse retina (columns). The size of the dot indicates the percentage of cells expressing the gene, and the color represents the average expression level of the gene in the indicated cell types.

**B.** Bar plots showing the GO terms enriched for the downregulated IR-DEGs (left) and upregulated EMPA-DEGs (right) of overall retinal cells. The color from light to dark indicates the statistical significance value from low to high.

**C.** GSEA of EMPA-DEGs mapping to GO pathway neuron death.

**D.** GSEA of IR-DEGs mapping to GO pathway neuron death.

**
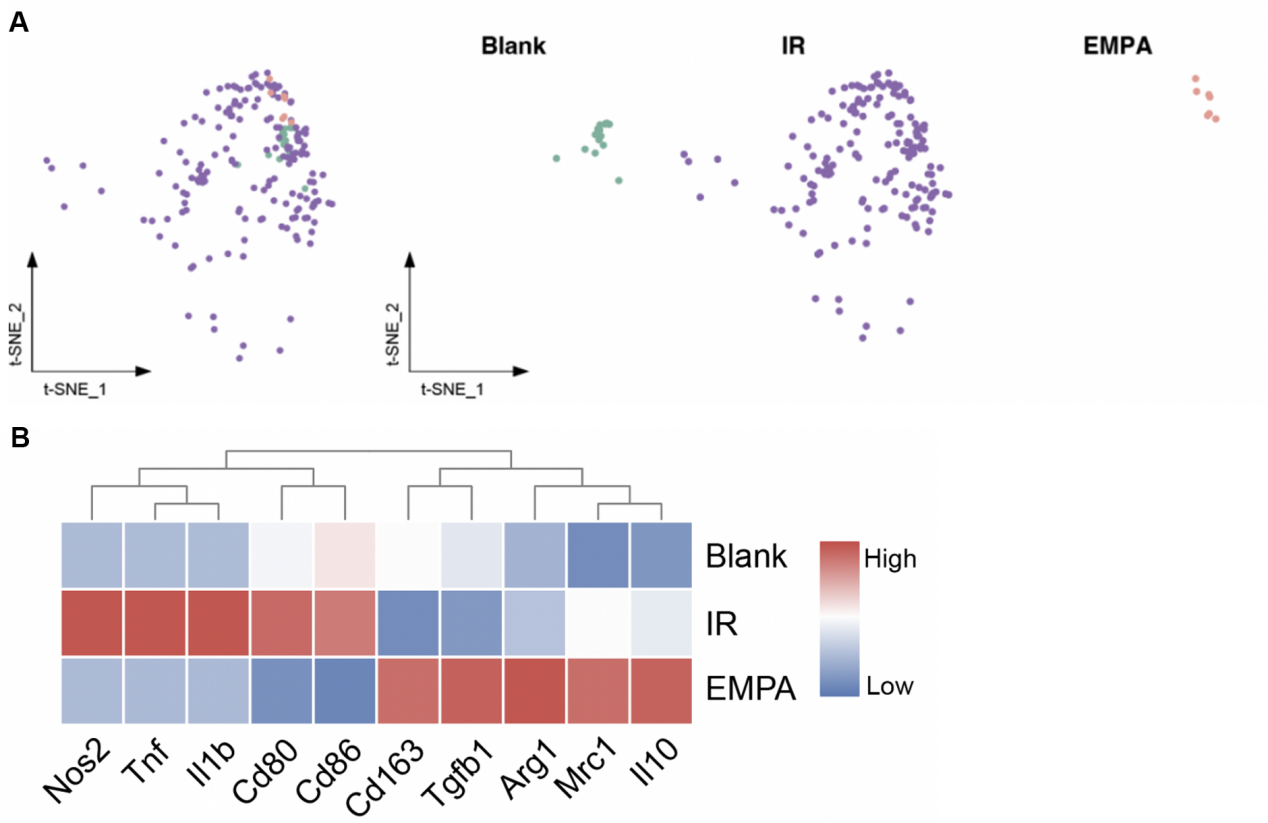
**

**Fig. S2. EMPA suppresses microglial activation in the IR retina**

1. t-SNE plot showing the distribution of microglia from three groups.
2. Heatmap showing the expression of M1 and M2 markers from three groups in microglia. The color key from blue to red indicates the expression level from low to high.


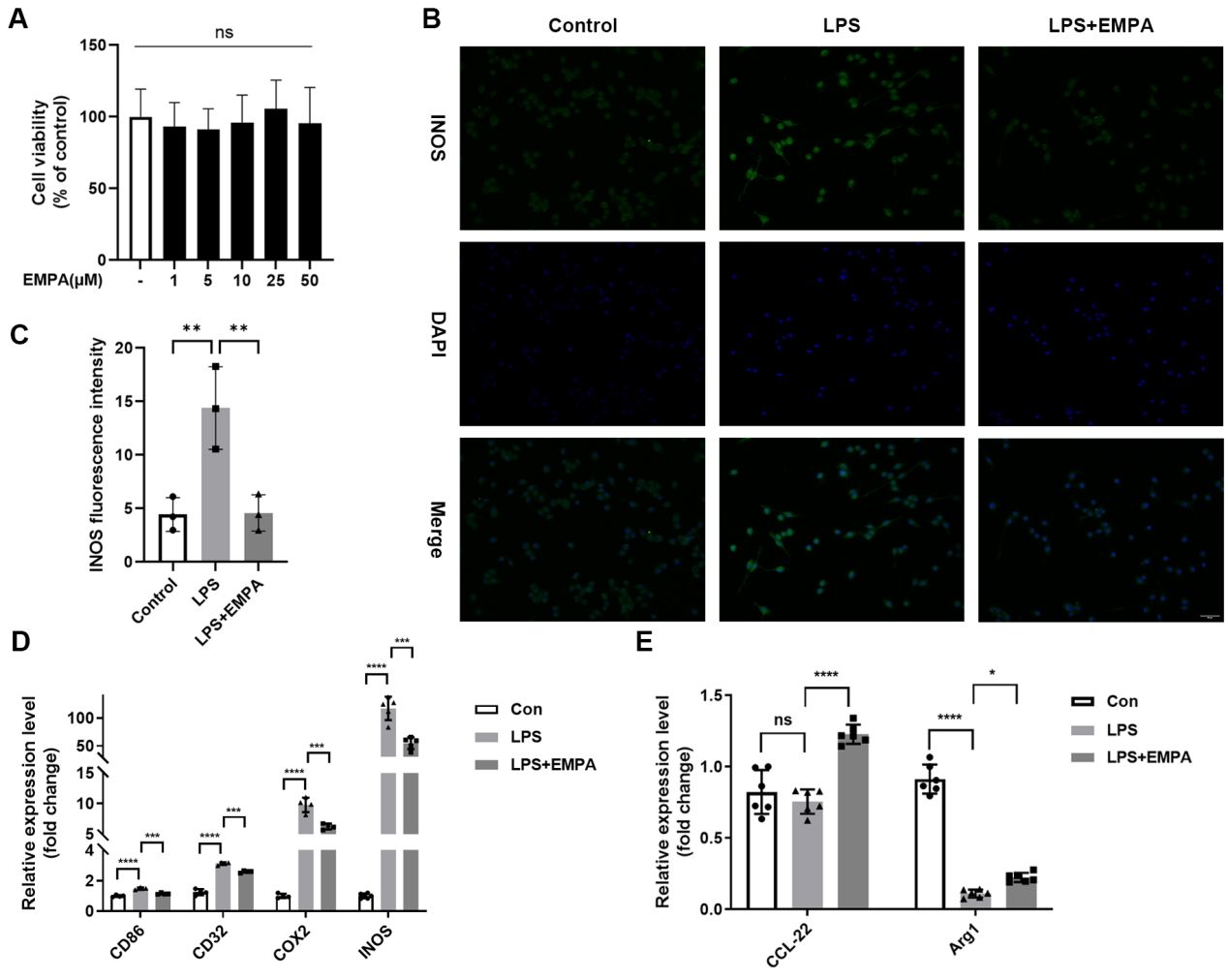


**Fig. S3. EMPA modulates LPS-induced polarization in BV2 cells treated with LPS**

**A.** BV2 cells were treated with indicated concentrations of EMPA. Cell viability was measured by CCK-8 assay.

**B.** Representative immunofluorescence images of INOS in control and LPS-stimulated BV2 cells treated with or without EMPA. Scale bar: 50 µm.

**C.** The quantity of the intensity of INOS immunofluorescence in BV2 cells (n=3).

**D-E.** qRT-PCR analysis of mRNA expression levels of M1 markers (CD68, CD32, COX2, INOS) and M2 markers (CCL-22, Arg1) (n=4-6).

**
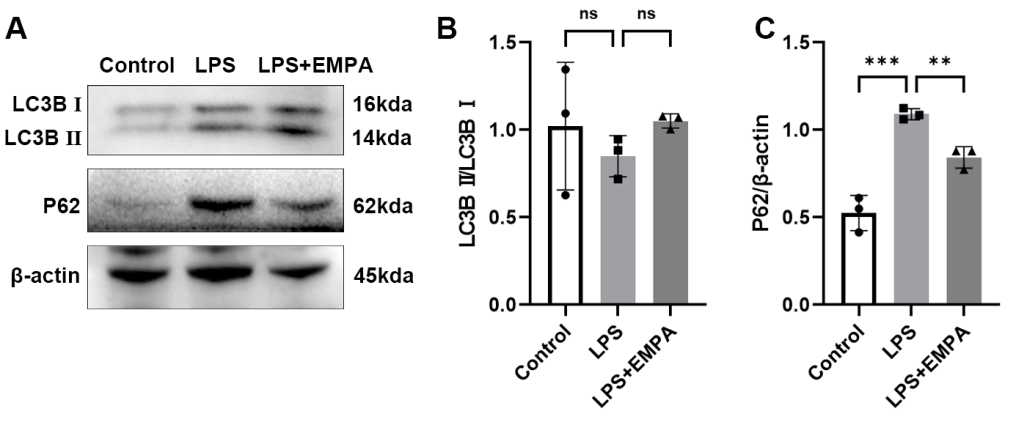
**

**Fig. S4. EMPA activates mitophagy in BV2 cells treated with LPS**

**A.** The levels of mitophagy-related proteins (LC3B and P62 ) were analyzed through Western blots. β-actin served as the internal protein control.

**B.** Relative densitometry quantitation of relative protein expression levels in the 3 groups of BV2 cells (n=3).


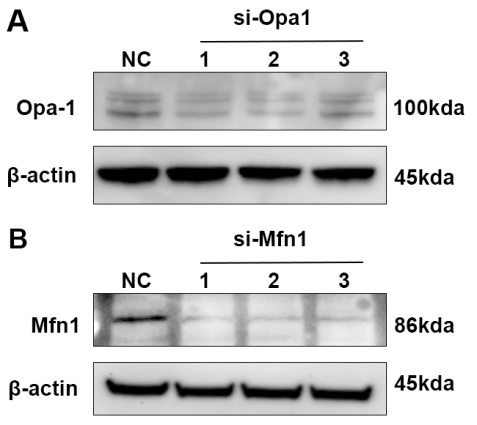


**Fig. S5. Knockdown efficiency of Opa1 (A) or Mfn1 (B) si RNA**

**Table S1. Primary antibodies and dilutions**

| Name | Company | Cat.No | Host | Dilution |
| --- | --- | --- | --- | --- |
| RBPMS | GeneTex | GTX118619 | Rb | IF(1:500) |
| TUJ1 | BioLegend | 801202 | Ms | IF(1:500) |
| Iba1 | Wako | 019-19741 | Rb | IF(1:500) |
| INOS | GeneTex | GTX130246 | Rb | IF(1:200) |
| Iba1 | Abcam | ab283319 | Ms | WB(1:1000) |
| β-actin | CST | 4970 | Rb | WB(1:1000) |
| GAPDH | CST | 2118 | Rb | WB(1:1000) |
| S100a9 | CST | 73425 | Rb | WB(1:1000) |
| NLRP3 | CST | 15101 | Rb | WB(1:1000) |
| ASC | Abclonal | A1170 | Rb | WB(1:500) |
| Caspase1 | Proteintech | 22915-1-AP | Rb | WB(1:1000) |
| IL-1β | Wanleibio | WL00891 | Rb | WB(1:500) |
| IL-18 | Wanleibio | WL01127 | Rb | WB(1:1000) |
| Mfn1 | Proteintech | 13798-1-AP | Rb | WB(1:1000) |
| Opa1 | Abcam | ab157457 | Rb | WB(1:1000) |
| Drp1 | Abcam | ab184247 | Rb | WB(1:1000) |
| LC3B | CST | 83506 | Ms | WB(1:1000) |
| P62 | CST | 23214 | Rb | WB(1:1000) |

**Table S2. qRT-PCR primer sequences**

| Gene | Forward | Reverse |
| --- | --- | --- |
| IL-1β | GCAACTGTTCCTGAACTCAACT | ATCTTTTGGGGTCCGTCAACT |
| IL-6 | AGTCCTTCCTACCCCAATTTCC | TTGGTCCTTAGCCACTCCTTC |
| TNF-α | CAGGCGGTGCCTATGTCTC | CGATCACCCCGAAGTTCAGTAG |
| CD86 | TCAATGGGACTGCATATCTGCC | GCCAAAATACTACCAGCTCACT |
| CD32 | AATCCTGCCGTTCCTACTGATC | GTGTCACCGTGTCTTCCTTGAG |
| COX2 | TGCACTATGGTTACAAAAGCTGG | TCAGGAAGCTCCTTATTTCCCTT |
| INOS | GGTGAAGGGACTGAGCTGTT | ACGTTCGTTCTCTTGCA |
| CCL-22 | CTGATGCAGGTCCCTATGGT | GCAGGATTTTGAGGTCCAGA |
| Arg1 | TCACCTGAGCTTTGATGTCG | CTGAAAGGAGCCCTGTCTTG |
